# Bile salt dependent lipase promotes intestinal adaptation in rats with massive small bowel resection

**DOI:** 10.1042/BSR20180077

**Published:** 2018-05-28

**Authors:** Yi Yang, Tao Zheng, Jiefei Zhou, Huanlei Song, Wei Cai, Linxi Qian

**Affiliations:** 1 Shanghai Institute for Pediatric Research, Xinhua Hospital, School of Medicine, Shanghai Jiao Tong University, 1665 Kongjiang Road, Shanghai 200092, China; 2 Shanghai Key Laboratory of Pediatric Gastroenterology and Nutrition, Xinhua Hospital, School of Medicine, Shanghai Jiao Tong University, 1665 Kongjiang Road, Shanghai 200092, China; 3 Department of Obstetrics, Xinhua Hospital, School of Medicine, Shanghai Jiao Tong University, 1665 Kongjiang Road, Shanghai 200092, China

**Keywords:** bile salt dependent lipase, intestinal adaptation, intestinal barrier, short bowel syndrome, Wnt/beta-catenin signaling

## Abstract

Intestinal adaptation is important for the short bowel syndrome (SBS) patients. Growing evidence has suggested that bile salt dependent lipase (BSDL) not only has the lipolytic activity, but also the immune-modulating and pro-proliferative activities. The purpose of the present study was to investigate the effects of BSDL on intestinal adaptive growth and gut barrier function in a rat model of SBS. Twenty-four male Sprague–Dawley rats were randomly divided into three experimental groups: sham group (rats underwent bowel transection and re-anastomosis), SBS group (rats underwent 80% bowel resection), SBS-BSDL group (SBS rats orally administered BSDL). The animals were weighed daily. The intestinal morpho-histochemical changes and intestinal barrier function were determined 14 days after the operations. Meanwhile, the expressions of Wnt signaling molecules in enterocytes were also analyzed by immunohistochemistry and Western blot. The postoperative weight gain was faster in the SBS rats treated with BSDL than in the SBS/untreated group. The SBS rats treated with BSDL had significantly greater villus height, crypt depth, and enterocyte proliferation in their residual intestines, as compared with the SBS/untreated group. The recovery of intestinal barrier function was promoted and the expressions of tight-junction proteins were increased in the SBS rats treated with BSDL. Additionally, the data indicated that the proadaptive activities of BSDL might be mediated by Wnt signaling activation in the enterocytes. These observations suggested that enteral BSDL administration promoted intestinal adaptive growth and barrier repairing by activating Wnt signaling pathway in SBS rats.

## Introduction

Short bowel syndrome (SBS) is defined as a malabsorptive state that results from a decreased gut absorptive area following a massive small bowel resection (mSBR) [1–3]. SBS leads to insufficient nutrient absorption and significant weight loss, and patients must depend on parenteral nutrition (PN) to meet nutritional requirements [4]. Although PN can improve the survival of SBS patients, the intestinal adaptation of the remaining bowel segment is still the primary determinant of decreasing malabsorption and malnutrition caused by SBS [5]. According to the literature, several bioactive agents might help to accelerate this adaptive process [6–8]. Consequently, interventions directed toward improving intestinal adaptation in SBS patients are of immense therapeutic interest.

Bile salt dependent lipase (BSDL) is an LeX-carrying glycoprotein secreted by the pancreas and activated by bile salts in the intestine [9]. BSDL is also expressed by the mammary gland and is present in human milk at a concentration between 100 and 200 μg/ml [10,11]. Once activated by bile salts in the duodenal lumen, BSDL, in concert with other pancreatic lipolytic enzymes and preduodenal lipase, acts to complete the digestion of dietary lipids [12,13]. In patients suffering from mSBR, the loss of absorptive surface area, the decrease in bile acid pool [14,15], and the reduction in pancreatic lipase secretion [16] result in inefficient fat absorption and steatorrhea. Therefore, patients with SBS have been found to benefit from low-fat diets early in the course of their treatment [17]. However, the long-chain fatty acids released from dietary fats are also some of the major trophic nutrients that stimulate intestinal adaptation [18,19]. Thus, we hypothesized that the enteral supplementation of BSDL after mSBR might contribute to fat absorption and promote intestinal adaptation in SBS patients.

Studies conducted over the past two decades have repeatedly shown that BSDL can not only hydrolyze dietary lipids, but also promote the proliferation of different cell types [20–22]. However, no signaling pathway has been directly indicated in the proliferative effect of BSDL. It is well known that various signaling cascades have been implicated in the control of intestinal adaptation, which is characterized by massive enterocyte proliferation. A possible role of Wnt signaling was suggested by a previous study, in which mSBR mice were found to have enhanced villus growth and enterocyte proliferation, as well as elevated intestinal expression of β-catenin [23]. The canonical Wnt signaling pathway regulates cell fate and proliferation, and that signaling involves a series of phosphorylation events that eventually result in the stabilization and translocation of β-catenin into the nucleus. The Axin/GSK-3β/CK-1/APC ‘destruction complex’ normally phosphorylates cytoplasmic β-catenin at specific N-terminal sites, Ser^33^, Ser^37^, and Thr^41^ [24], which leads to the ubiquitin-mediated degradation of this protein [25]. The canonical Wnt signaling allows the phosphorylation of GSK-3β and inhibits the ‘destruction complex’, which results in cytoplasmic β-catenin stabilization [26]. After the entry of BSDL into the intestinal lumen, the enzymatically active BSDL can be endocytosed by enterocytes, and then released at the basolateral membrane of the cell [27]. As revealed in *Drosophila*, the endocytosis process is essential for Wnt/Wingless signaling transduction, the inhibition of endocytosis caused a reduction in this signaling [28]. Furthermore, the activation of Wnt signaling by LiCl, an inhibitor of GSK-3β, was reported to be prevented by inhibitors of endocytosis [29]. All these clues prompted us to think whether the endocytosis of BSDL has some relationship with Wnt signaling activation. Notably, a more direct evidence provided by Lombardo et al. showed that the non-secreted mutant form of BSDL can modulate Wnt/β-catenin signaling in pancreatic tumor cells [30].

The purpose of the present study was to investigate the effects of enteral BSDL administration on intestinal adaptive growth and barrier rehabilitation in a rat model of mSBR. Moreover, our unpublished research identified a phenomenon, in which BSDL could induce the nucleus translocation of cytoplasmic β-catenin in the cultured intestinal cells. Thus, it was of great interest to investigate whether BSDL could activate the Wnt signaling pathway in SBS rats.

## Materials and methods

### Animals

Young (4 weeks) male Sprague–Dawley rats were used. The rats were housed in individual cages in the animal care facility under controlled conditions of temperature (22–28°C) and humidity, with a 12-h light, 12-h dark cycle. The animals were given free access to water and standard pelleted rat food during the experiment. All procedures performed in studies involving animals were in accordance with the ethical standards. The protocol was approved by the Institutional Animal Care and Use Committee of Xinhua Hospital.

### Experimental design

A total of 24 rats (130–140 g) were randomly assigned into three groups (*n*=8 per group): the sham group, the SBS group, and the SBS-BSDL group. Animals were fasted for 16 h prior to laparotomy, and intestinal surgery was performed as previously described [31]. Briefly, the operation was performed under anesthesia by intraperitoneal injection of pentobarbital sodium at the dosage of 40 mg/kg body weight. During the surgery, the abdomen was opened by a midline incision. The ligament of Treitz and the ileal–cecal junction was identified and marked. For SBS rats, enterectomy was performed by removing approximately 80% of the small intestine, leaving approximately 10 cm of the terminal ileum and 5 cm of the proximal jejunum. The continuity of the remaining intestine was restored by end-to-end anastomosis. For sham control rats, all surgical manipulations were performed the same as above, except for the resection procedure. All animals received a fluid resuscitation by intraperitoneal injection of saline (10 ml of 0.9% NaCl) before the abdominal wall was closed. The SBS-BSDL group rats underwent bowel resection as SBS rats and were administered recombinant BSDL by oral gavage.

After their operations, the rats were transferred back to their individual cages, fasted for 24 h following laparotomy, and then fed a regular diet from day 2. Every morning, from days 1 to 14, each rat in the SBS-BSDL group was administered BSDL at a dosage of 0.04 g/kg body weight by oral gavage. The preparation of recombinant BSDL protein was described in Supplementary Materials and Methods. The dosage of BSDL applied in this experiment was calculated according to the human milk BSDL concentration (200 μg/ml), assuming that each rat drinks 200 ml.kg^−1^.day^−1^ milk. Body weight and food intake were recorded everyday, and BSDL dosage was adjusted according to the change in body weight. The sham and SBS groups were given 1 ml warm water by oral gavage every morning. The rats were killed on day 14 by decapitation under anesthesia. Before being killed, all the rats were treated with 2 ml of 50 mg/ml d-xylose by oral gavage. The required organs and blood samples were collected after killing. The jejunum and ileum segments of each rat were collected at the same site of the bowel.

### Bacterial translocation

The mesenteric lymph nodes (MLN) of the ileocecal region, liver, and spleen samples were collected under sterile conditions. The specimens were diluted in PBS (0.1 ml per 0.1 g) and homogenized, and 100 μl suspension was plated on MacConkey and Whole Blood Agar. After 24 h of culture under appropriate conditions (aerobic, at 37°C), the positive bacterial colonies were counted. A positive result was determined as ten or more clones found on the plate and was considered indicative of bacterial translocation (BT) [32]. The results were described as the percentage of animals exhibiting BT.

### Determination of intestinal permeability

Blood samples (3 ml) were collected with anticoagulant, and then centrifuged at 3000 rpm for 10 min. Later, the supernatants were transferred into a new microcentrifuge tube. The pre-treated samples were analyzed using the d-xylose assay kit (Nanjing Jiancheng Company). The results were expressed as the d-xylose concentration of serum (mmol/l).

### Histological analysis

Pieces of small intestine samples were first rinsed in cold normal saline and then immediately fixed in 4% paraformaldehyde (pH 7.2). After 72 h of fixation, the tissue was embedded in paraffin wax on the oriented edge and cut into 5-μm-thick sections for Hematoxylin and Eosin (H&E) staining. The villus height and crypt depth of ten vertically well-orientated villi and crypts were determined using an analysis system (NIS-elements suite; Nikon) by experienced observers who were unaware of treatment group (T.Z. and H.S.). Villus height was determined from the crypt opening to the top of the villus and crypt depth from the base of the crypt to its opening using ocular micrometers.

### Immunohistochemistry (immunofluorescence) staining

Five micrometers thick paraffin-embedded sections of ileum were prepared as above. Sections were treated with 0.3% hydrogen peroxide in methanol for 20 min at room temperature to quench the endogenous peroxidase activity. After washing with PBS, the sections were placed in citrate buffer (pH 6.0) and subjected to antigen retrieval by microwave heating for 20 min. Then, the sections were immunostained using the ABC kit (Boster). After blocking with 5% BSA at room temperature for 20 min, sections were incubated overnight at 4°C with the following primary antibodies: rabbit anti-Cyclin-D1 (2978, Cell Signaling Technology), rabbit anti-cleaved caspase3 (9661, Cell Signaling Technology), rabbit anti-p-Ser^552^-β-catenin (5651, Cell Signaling Technology), and rabbit anti-non-p-Ser^33/37^/Thr^41^-β-catenin (8814, Cell Signaling Technology). Control sections were incubated with normal rabbit IgG at the same dilution as the primary antibody. The sections were incubated with biotinylated secondary antibody for 20 min at 37°C. After washing with PBS, sections were treated with avidin–biotin–peroxidase complex for 20 min at 37°C according to the manufacturer’s instructions. Then, 3-diaminobenzidine (DAB) was added as a chromogen to stain each section. Mayer’s Hematoxylin was used for nuclear counterstaining.

For immunofluorescence staining, the sections were hydrated in PBS for 15 min, blocked for 1 h with 5% BSA, and incubated overnight at 4°C with the following primary antibodies: rabbit anti-LGR5 (AP2745d, Abgent) and rabbit anti-claudin-1 primary antibodies (ab15098, Abcam). Control sections were incubated with normal rabbit IgG at the same dilution as the primary antibody. After rinsing with PBS, the slides were incubated for 1 h with FITC goat anti-rabbit IgG. The sections were mounted with DAPI-containing medium (Vector Laboratories) for counterstaining of nuclei. The stained sections were observed by florescent microscope (Nikon Eclipse 80i). Slides were analyzed and assessed by blinded investigators (T.Z. and H.S.).

### Western blotting analysis

The ileum mucosa samples were homogenized in cold RIPA buffer (PBS, 1% NP-40, 0.5% sodium deoxycholate, 0.1% SDS, 1 μg/ml PMSF, 1.0 mM sodium orthovanadate, 1× mammalian protease inhibitor cocktail; Sigma-Aldrich). Total protein was quantitated using BCA assay reagent (Pierce). Aliquots (50 μg total proteins) were loaded into SDS/polyacrylamide gels and transferred on to a PVDF-Plus membrane. After blocking in 5% fat-free milk, the membranes were incubated overnight at 4°C with the following primary antibodies: rabbit anti-claudin-1 (ab15098, Abcam), rabbit anti-occludin (ab216327, Abcam), rabbit anti-β-catenin (8480, Cell Signaling Technology), rabbit anti-p-Ser^552^-β-catenin (5651, Cell Signaling Technology), rabbit anti-non-p-Ser^33/37^/Thr^41^-β-catenin (8814, Cell Signaling Technology), rabbit anti-GSK-3β (9315, Cell Signaling Technology), rabbit anti-Ser^9^-GSK-3β (5558, Cell Signaling Technology) and rabbit anti-GAPDH (2118, Cell Signaling Technology). The primary antibodies were detected with horseradish peroxidase-conjugated goat anti-rabbit IgG secondary antibody, followed by the use of a chemiluminescence system (Pierce) and ChemiDOC XRS+ imaging system (Bio-Rad). Protein expression was normalized to the same sample expression of GAPDH. Signals from the phosphorylated proteins were normalized to the amount of total target proteins. The relative band intensity was quantitated by ImageJ software (National Institutes of Health in United States).

### Statistics

Data are presented as the mean ± S.E.M. Two-tailed unpaired Student’s *t* test was used for comparisons of mean values between two groups. Differences between multigroups were analyzed by one-way ANOVA, followed by a Student–Newman–Keuls test. Fisher’s exact test was used for comparisons of BT frequency values between two groups. All statistical analyses were carried out using SPSS 13.0 software. *P*-value less than 0.05 was considered significant.

## Results

### Food intakes and body weight changes

**Figure 1:**
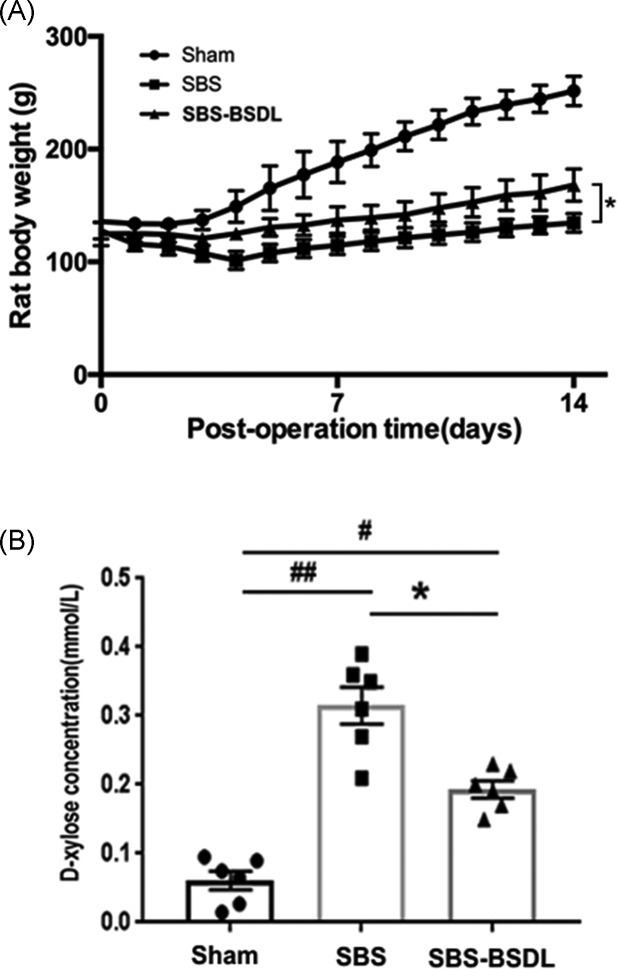
Body weight and intestinal permeability (**A**) Changes in the body weight of all rats during the post-operation days. Data are expressed as mean ± S.E.M. *P<0.05, as compared with the SBS group from days 4 to 14 post-operation. (**B**) Intestinal permeability expressed as the serum d-xylose concentration (mmol/l). Data are expressed as mean ± SEM; rats *n*=6 per group. *P<0.05, as compared with the SBS group. ^#^*P*<0.05, ^##^*P*<0.01, as compared with the sham group.

All rats in these groups survived throughout the entire experimental period and had similar initial mean body weights (130–140 g) on the day of surgery, as represented by day 0. During the 14-day study period, there were no significant differences in water and food intake amongst different group animals. Changes in mean body weight during the post-operation recovery period are illustrated in Figure 1A.

The rats in the sham group slightly lost weight during the initial 48 h following operation, and then gained weight from day 3. The rats in the SBS and SBS-BSDL groups showed a tendency to lose weight until day 4 post-operation. Four days after operation, the SBS and SBS-BSDL rats lost 27 and 11% of their pre-operative weight, respectively. From days 5 to 14 post-operation, their body weights steadily increased. The body weight of the rats in the SBS group was much lower than that in the sham group (*P*<0.05, for each time point following day 2), which could have been due to insufficient nutrient absorption and diarrhea after mSBR. Supplementation with BSDL led to an additional increase in body weight as compared with the SBS only rats (*P*<0.05, for each time point following day 3). The final weight of the rats in the SBS-BSDL group was 25% higher than that in the SBS group (SBS: 134.50 ± 8.38 compared with SBS-BSDL: 168.23 ± 14.42 g, *P*<0.05). Three rats from the SBS-BSDL group and six rats from the SBS group had visible diarrhea.

### BT

The BT rate of the SBS and SBS-BSDL groups was higher than that of the sham group. Only 12.5% sham rats exhibited BT to the MLN (level I) and none in either spleen or liver. In the SBS group, however, 100% BT to the MLN (level I), 75% BT to the spleen (level II), and 25% BT to the liver (level II) were detected, with significant difference as compared with the sham group (*P*<0.01 for MLN; *P*<0.05 for spleen). The SBS-BSDL rats exhibited a 25% BT to the MLN (level I), 12.5% BT to spleen (level II), and no translocation to the liver (level II). The BT frequency of SBS-BSDL rats was significantly lower than that of the SBS only rats (*P*<0.05 for MLN and spleen) (Table 1).

**Table 1:** Frequency of BT to the MLNs and peripheral organs (% of animals exhibiting BT)

	MLN (level I)	Spleen (level II)	Liver (level II)
Sham, *n*=8	1 (12.5%)	0	0
SBS, *n*=8	8 (100%)^‡^	6 (75%)^†^	2 (25%)
SBS-BSDL, *n*=8	2 (25%)*	1 (12.5%)*	0

Mice *n*=8 per group. Fisher’s exact test. *P<0.05, as compared with the SBS group. ^†^*P*<0.05. ^‡^*P*<0.01, as compared with the sham group.

### Intestinal permeability

To test whether BSDL treatment changed the intestinal permeability in the residual intestine, we carried out the d-xylose absorption assay before killing the animals (Figure 1B). We found that the SBS only rats had a significant increase in intestinal permeability, as measured by serum d-xylose concentration, as compared with the sham group (*P*<0.01). However, the administration of BSDL reduced the serum d-xylose concentration by 43%, as compared with the SBS group (SBS: 0.33 ± 0.04 compared with SBS-BSDL: 0.19 ± 0.02 mmol/l, *P*<0.05).

### Morphology of intestinal tissues

**Figure 2:**
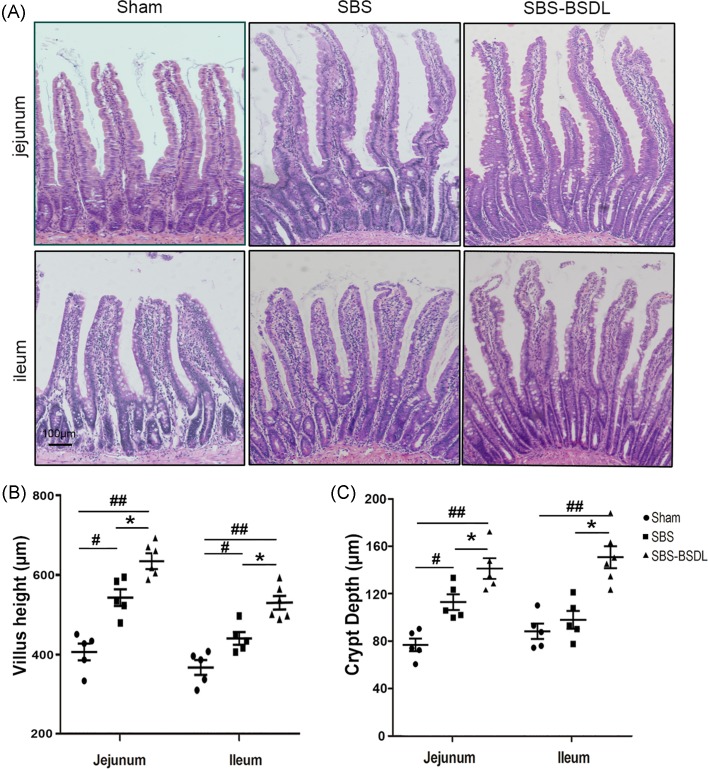
Villus and crypt architecture, as revealed by H&E staining (**A**) Jejunum and ileum of the sham group, the SBS group, and the SBS-BSDL group (crypt-to-villus longitudinal section, magnification 100×). (**B**) Quantitation of villus height of jejunum and ileum. (**C**) Quantitation of crypt depth of jejunum and ileum. Data are expressed as mean ± S.E.M.; rats *n*=5–6 per group. *P<0.05, as compared with the SBS group. ^#^*P*<0.05, ^##^*P*<0.01, as compared with the sham group.

Representative H&E-stained jejunal and ileal segments for the three independent groups are shown in Figure 2A. We observed a significant increase in jejunal and ileal villus height in the SBS only rats and SBS-BSDL rats, as compared with the sham control. mSBR resulted in a 0.37-fold increase in jejunal villus height (*P*<0.05), a 0.22-fold increase in ileal villus height (*P*<0.05), as compared with the sham control. Supplementation with BSDL led to an additional 0.23-fold increase in jejunal villus height (*P*<0.05), and an additional 0.27-fold increase in ileal villus height (*P*<0.05), as compared with the SBS only rats (Figure 2B).

We also observed an increase in jejunal and ileal crypt depth in the SBS only rats and SBS-BSDL rats, as compared with the sham control. Supplementation with BSDL led to an additional 0.34-fold increase in jejunal crypt depth (*P*<0.05), and an additional 0.47-fold increase in ileal crypt depth (*P*<0.05), as compared with the SBS only rats (Figure 2C).

### Tight junction protein expression in intestine

**Figure 3:**
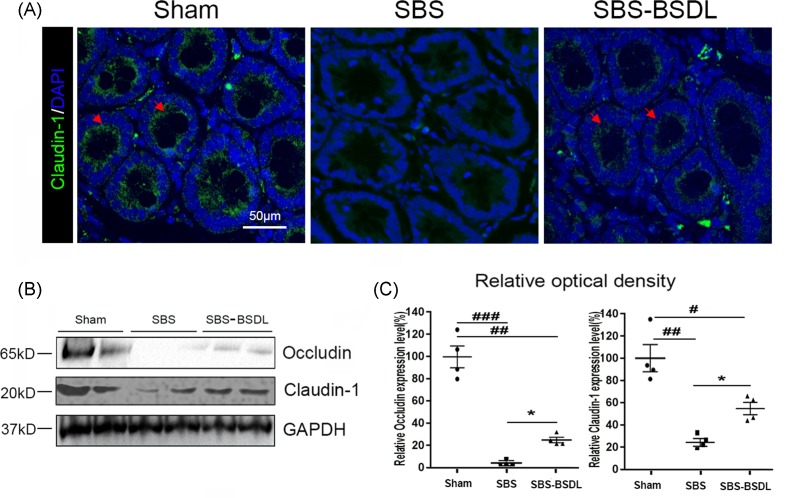
The expressions of TJ proteins in ileum (**A**) Representative claudin-1 immunofluorescence images in ileum of the sham group, the SBS group, and the SBS-BSDL group (crypt vertical section, magnification 200×). (**B**) Western blot representing total occludin and claudin-1 in ileum of the sham group, the SBS group, and the SBS-BSDL group. (**C**) Densitometric quantitation of relative expression levels normalized to GAPDH. Data are expressed as mean ± S.E.M.; rats *n*=4 per group. *P<0.05 as compared with the SBS group. ^#^*P*<0.05, ^##^*P*<0.01, ^###^*P*<0.001, as compared with the sham group.

Tight junction (TJ) proteins play a crucial role in intestinal barrier. Disruption of TJ proteins may lead to the increased permeability of intestinal barrier to the passage of luminal bacteria. In the present study, immunofluorescence was used to examine the distribution of claudin-1, an important TJ protein, in the ileum of three independent groups. The ileal section of sham rats showed that claudin-1 was expressed along the apical surface of crypt cells in linear pattern. However, no claudin-1 immunofluorescence signal was observed on the surface of crypt cells of SBS only rats. In contrast, the expression of claudin-1 protein on the surface of crypt cells was recovered in the SBS-BSDL rats, but the linear pattern was only partially reconstructed and appeared irregular at the apical surfaces (Figure 3A).

Western blot analysis revealed that the expression levels of occludin and claudin-1 in the residual ileum of SBS only rats were significantly reduced as compared with the sham controls (*P*<0.01). This reduction was partially reverted by oral administration of BSDL in the SBS-BSDL rats (*P*<0.05) (Figure 3B-C).

### Epithelial cell proliferation and apoptosis

**Figure 4:**
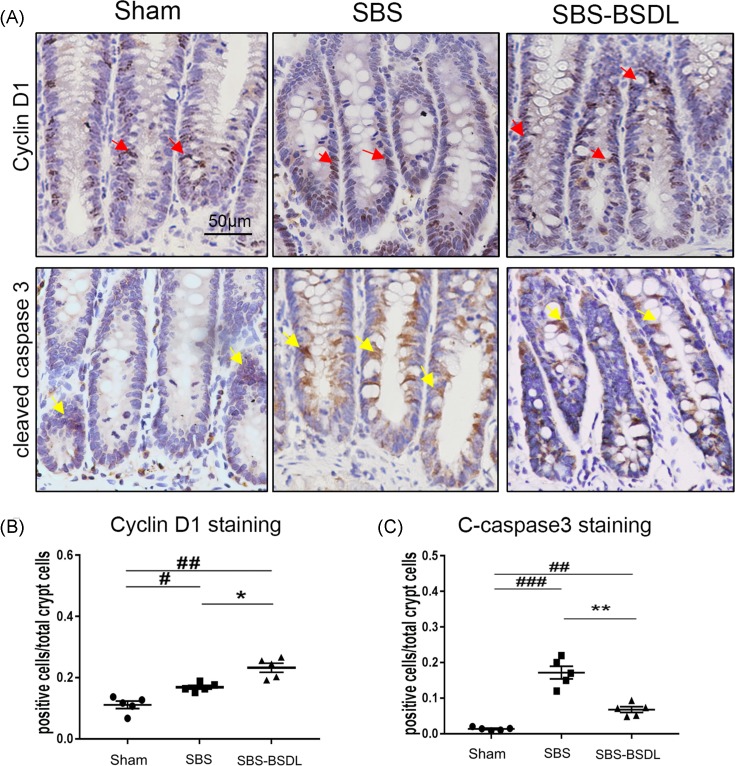
Cell proliferation and apoptosis in ileum (**A**) Representative Cyclin D1 and cleaved caspase 3 immunohistochemical images in ileum of the sham group, the SBS group, and the SBS-BSDL group (crypt longitudinal section, magnification 200×). Arrows indicate the positive cells. (**B**) The percentage of Cyclin D1-positive cells amongst total crypt cells. (**C**) The percentage of cleaved caspase 3-positive cells amongst total crypt cells. Data are expressed as mean ± S.E.M.; rats *n*=5 per group. *P<0.05, **P<0.01, as compared with the SBS group. ^#^*P*<0.05, ^##^*P*<0.01, ^###^*P*<0.001, as compared with the sham group.

Cyclin D1 is a key regulator of cell cycle progression. The activation of the Cyclin D1 expression has been associated with the proliferation of intestinal crypt cells [33]. To evaluate the proliferation of intestinal epithelial cells, we analyzed the Cyclin D1 expression in the ileum for the three independent groups. The representative Cyclin D1-stained sections are shown in Figure 4A (upper panels). The SBS group demonstrated a significant increase in the proportion of Cyclin D1-positive cells (expressed as the percentage of positive cells amongst total crypt cells), as compared with the sham group (*P*<0.05). BSDL administration additionally increased the proportion of Cyclin D1-positive cells in the residual ileum, as compared with the SBS only rats (Figure 4B) (*P*<0.05).

We also detected cell apoptosis in the ileal sections through cleaved caspase 3 immunostaining, and the results are shown in Figure 4A (lower panels). The proportion of cleaved caspase 3-positive cells in SBS group (expressed as the percentage of positive cells amongst total crypt cells) was greater than that in the control group (*P*<0.001). BSDL administration resulted in a significant reduction in apoptotic cells in the residual ileum, as compared with the SBS only rats (Figure 4C) (*P*<0.01).

### Wnt/β-catenin signaling activation in intestine *z*

β-catenin plays a central role in intestinal proliferation, which is mainly expressed in the cell membrane [34]. Phosphorylation of β-catenin at Ser^552^ is associated with the nuclear import of β-catenin. GSK-3β phosphorylation at Ser^9^, and subsequent low phosphorylation status of β-catenin at Ser^33/37^/Thr^41^ are associated with the protection of β-catenin from proteosomal degradation [35].

**Figure 5:**
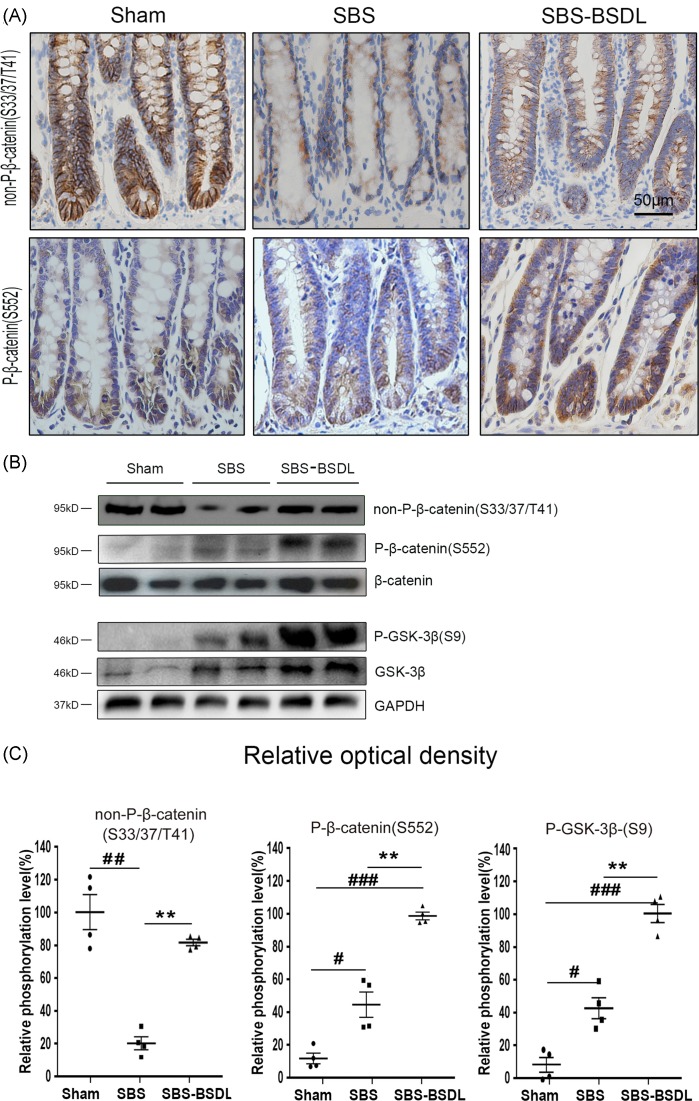
The expressions of the Wnt/β-catenin pathway proteins in ileum (**A**) Representative non-P-β-catenin (Ser^33/37^/Thr^41^) and P-β-catenin (Ser^552^) immunohistochemical images in ileum of the sham group, the SBS group, and the SBS-BSDL group (crypt longitudinal section, magnification 200×). (**B**) Western blot representing total β-catenin, total GSK-3β, non-P-β-catenin (Ser^33/37^/Thr^41^), P-β-catenin (Ser^552^), and GSK-3β (Ser^9^) in ileum of the sham group, the SBS group, and the SBS-BSDL group. (**C**) Densitometric quantitation of phosphorylation levels normalized to their respective total proteins. Data are expressed as mean ± S.E.M.; rats *n*=4 per group. **P<0.01, as compared with the SBS group. ^#^*P*<0.05, ^##^*P*<0.01, ^###^*P*<0.001, as compared with the sham group.

To assess whether Wnt/β-catenin signaling is involved in the intestinal adaptation enhanced by BSDL treatment, we examined the protein level of active non-P-β-catenin (Ser^33/37^/Thr^41^) in the ileum by immunohistochemistry. The representative sections are shown in Figure 5A (upper panels). We found that the abundance of active non-P-β-catenin (Ser^33/37^/Thr^41^) was reduced in the SBS only rats as compared with the sham rats, indicative of enhanced β-catenin degradation during intestinal adaption. BSDL administration reverted the abundance of active non-P-β-catenin (Ser^33/37^/Thr^41^) in rat residual ileum, as compared with the SBS only rats. Moreover, the expression level of P-β-catenin (Ser^552^) in the ileum was also examined by immunohistochemistry. The representative sections are shown in Figure 5A (lower panels). We found that the SBS group showed an increase in the abundance of P-β-catenin (Ser^552^) in the intestinal crypts as compared with the sham group, which indicated an increase in β-catenin nuclear translocation during intestinal adaption. BSDL administration additionally increased the abundance of P-β-catenin (Ser^552^) in the crypts, as compared with the SBS only rats.

Western blot analysis was used to confirm the changes in Wnt/β-catenin pathway protein expression levels (Figure 5B). The expression level of total β-catenin was unchanged within three experimental groups. However, the total GSK-3β accumulation was increased in the SBS and SBS-BSDL groups as compared with the sham group. The phosphorylation analysis showed significantly higher expression levels of P-GSK-3β (Ser^9^), non-P-β-catenin (Ser^33/37^/Thr^41^), and P-β-catenin (Ser^552^) in the SBS-BSDL group as compared with those in the SBS group (*P*<0.01 for each protein) (Figure 5C).

### LGR5-positive stem cells in intestine

**Figure 6:**
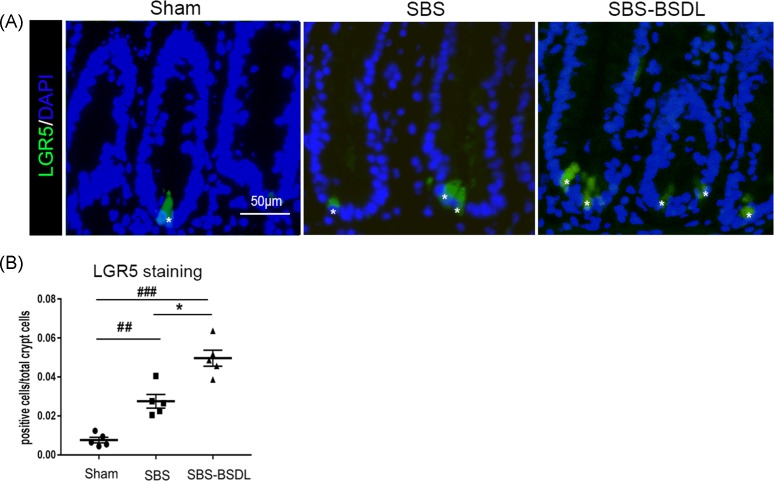
LGR5 positive stem cells in ileum (**A**) Representative LGR5 immunofluorescence images in ileum of the sham group, the SBS group, and the SBS-BSDL group (crypt longitudinal section, magnification 400×). Asterisks indicate the positive cells. (**B**) The percentage of LGR5-positive cells amongst total crypt cells. Data are expressed as mean ± S.E.M.; rats *n*=5 per group. *P<0.05, as compared with the SBS group. ^##^*P*<0.01, ^###^*P*<0.001, as compared with the sham group.

LGR5 has been identified as a target of Wnt signaling and is expressed in the intestinal stem cells (ISCs) at the crypt base [36,37]. Representative LGR5-stained sections for the three independent groups are shown in Figure 6A. The ISCs are the crypt base columnar cells that were strongly stained by LGR5, and the expansion of these cells is responsible for the intestinal regeneration after injury [38]. In the sham group, the sections showed that the proportion of LGR5-positive cells amongst crypt cells was low due to the limited injury. The proportion of 0.8 ± 0.14% LGR5-positive cells amongst crypt cells, which accounted for approximately one positive cell per crypt, in the sham group is close to the value revealed by Cosentino et al. [39] in the normal mice intestine. In the SBS rats, the proportion of LGR5-positive cells (expressed as the percentage of positive cells amongst total crypt cells) was increased as compared with the sham rats (*P*<0.01). BSDL administration resulted in an additional increase in the proportion of LGR5-positive cells in the residual ileum, as compared with the SBS only rats (*P*<0.05) (Figure 6B).

## Discussion

BSDL is distinguished from other esterases by its dependence on bile salts and its presence in breast milk [40,41]. The physiological role of BSDL in breast milk has been studied for decades, and its therapeutic potential for preterm birth was recently recognized. Wang et al. [42] reported that the increased level of BSDL in milk reduced the mortality and improved the growth of premature mice. In 2014, one prospective phase 2 clinical trial revealed that BSDL supplementation significantly improved the growth velocity and increased the absorption of long-chain polyunsaturated fatty acids in preterm infants [43]. Although not observed in the full enrolled population in phase 3 trial, the positive effect of BSDL on growth velocity was seen in small for gestational age infants [,^44^]. It indicated that oral BSDL administration for infants may be selectively beneficial for some specific subpopulation, and maybe the population with specific diseases. The SBS infants suffer from severe weight loss and diarrhea. In the treatment of SBS, the weight gain velocity is considered to be a crucial index used to evaluate the effects of enteral and PN [45]. The improvement of fat digestion and/or absorption is beneficial for the patients to increase the weight gain velocity and consequently contributes to the long-term outcomes [46]. The BSDL is able to hydrolyze a broad variety of lipids, and the supplementation of BSDL may benefit the SBS patients by improving lipid digestion and/or absorption. Thus, we investigated the effect of enteral BSDL administration on the recovery of the rats following mSBR surgery. Our results indicated that BSDL has positive effect on body weight gain and intestinal healing in the rat model of SBS. Moreover, the present study also identified a novel mechanism, other than the nutritional one, which is underlying the beneficial effect of BSDL.

Following mSBR, the remaining bowel undergoes a pattern of well-described morphological adaptation, which begins within 24–48 h of the operation. Similar to previous studies [47,48], the present study demonstrated that mSBR resulted in increased villus height and crypt depth, accompanied by an increase in epithelium proliferation. The increased villus height and crypt depth might cause an increased absorptive surface area and the enhancement of the absorption ability during the intestinal adaptation of rats following mSBR. The epithelium proliferation. We also observed an augment of enterocyte apoptosis in the rats following mSBR. The enterocyte apoptosis after mSBR is known to accompany the proliferative changes [49]. Intriguingly, BSDL treatment appears to co-ordinate the proliferation and apoptosis in the enterocytes and foster a new homeostasis, which enhances the intestinal growth. Howles et al. [21] found that the depletion of BSDL activity can cause fat-derived intestinal injury in neonatal mice. However, there was no study on whether direct oral BSDL administration can have effect on intestinal injury. Our study first showed that oral BSDL administration is able to protect against SBS-induced intestinal injury by increasing proliferation and decreasing apoptosis of enterocytes.

The intestinal barrier dysfunction is prevalent in SBS, and is manifested by translocation of luminal microbes to MLN and, subsequently, to peripheral organs such as the liver and spleen [49,50]. Our study showed that the SBS group exhibited much higher BT rates than the sham group, while the BT rates detected in the SBS-BSDL group were lower than those in the SBS group. These findings indicated that BSDL had potential protective effects on BT that occurred in SBS. Multiple factors might contribute to the enteric BT in SBS, including gut bacterial overgrowth and impairment of gut-associated anatomical and immune barriers. Previous studies showed that undigested lipids in the intestine could cause gut bacterial overgrowth and impede the adaptation process in SBS patients [51]. The oral administration of BSDL can speed up lipid digestion and prevent undigested lipids from accumulating in the gut [21]. The TJ proteins form a seal between adjacent epithelial cells near the apical surface, thus preventing the paracellular diffusion of microorganism across the epithelium [52]. In our study, the reverted TJ protein expression was microscopically revealed in the intestinal tissue of the SBS-BSDL rats, as compared with the SBS only rats, and these changes were confirmed by Western blot. Until now, there have been no reports on the impact of BSDL on TJ protein expression. Our study showed that BSDL could strengthen the intestinal barrier by up-regulating the expression of TJ proteins. Nevertheless, further studies focussing on the immune barriers should be conducted.

Growing evidence has suggested that the Wnt/β-catenin signaling cascade is implicated in the control of stem cell activity, cell proliferation, and cell survival during regeneration of the gastrointestinal epithelium [53]. Besides studying the proadaptive effect of BSDL in the rat model of SBS, we tried to gain further insight into the mechanism of BSDL on intestinal adaptation. Hence, we analyzed the expression and phosphorylation of some components of the Wnt signal transduction pathway. β-catenin, a crucial downstream effector of the Wnt signaling pathway, was previously shown to have an increased expression in the intestinal epithelium of SBS rats [23]. However, in another study, the level of *β-catenin* mRNA was found to significantly decrease in SBS rats, as revealed by microarray assay [54]. The inconsistency between these two studies prompted us to investigate further, by analyzing not only the total protein level of β-catenin, but also the phosphorylation of this protein. In the present study, we observed that there was no significant difference in the total β-catenin expression level between the SBS only rats and the sham control. However, the phosphorylation of β-catenin at Ser^552^, which is associated with the nuclear import of β-catenin [35], was increased in SBS rats. GSK-3β is another essential effector in the Wnt/β-catenin pathway. GSK-3β phosphorylation at Ser^9^ leads to its decreased activity of phosphorylating β-catenin at Ser^33/37^/Thr^41^, and in turn, protects β-catenin from proteosomal degradation [35]. We found that the bowel resection increased both the total protein of GSK-3β and its phosphorylated form at Ser^9^. However, we also found that the active form of non-P-β-catenin (Ser^33/37^/Thr^41^) was decreased in the SBS only rats, as revealed by both immunohistochemistry and immunoblot. This result suggested that the phosphorylation of P-β-catenin at Ser^33/37^/Thr^41^ may be modulated not only by GSK-3β but also by other kinases, such as cyclin-dependent kinase 2 [55]. Taken together, these data indicated that although Wnt/β-catenin signaling is implicated in the SBS intestinal adaptation, its activation is complex during this process. In the present study, the BSDL treatment was demonstrated to induce even higher phosphorylation level of β-catenin at Ser^552^ and GSK-3β at Ser^9^, and reverted phosphorylation level of β-catenin at Ser^33/37^/Thr^41^ in the intestinal tissue of SBS rats. These data provided evidence that oral administration of BSDL could additionally activate the Wnt/β-catenin signaling during intestinal adaptation.

In small intestine, the epithelial lining is rapidly regenerated every 4–5 days by continuous proliferation of ISCs residing near the base of crypts [56]. Previous studies demonstrated that the population of ISCs expanded following mSBR in the models of mice and zebrafish [57,58]. The mouse mSBR model provided evidence that early expansion of ISCs and subsequent crypt fission contributed to sustained long-term adaptive growth in residual intestine [57]. However, the morphometric changes, including villus height and crypt depth, following mSBR were unsustainable and would return to the sham levels by 16 weeks post-resection [57]. Therefore, therapies that promote expansion of these cells might result in sustained benefits for patients with intestinal failure caused by various factors [59]. The administration of Teduglutide (a GLP-2 analog) has previously been shown to increase the number of ISCs and the percentage of surviving crypts in radiation-induced intestinal failure [60]. In the present study, we used LGR5 to evaluate the effects of BSDL on the expansion of ISCs in the mSBR rats. LGR5 is specifically expressed on the crypt base columnar cells and can be used as a biomarker of ISCs. It was reported that the expression of LGR5 protein could accelerate the cell cycle via Wnt/β-catenin pathway [61]. We observed that the proportion of LGR5-positive ISCs was increased in the intestinal crypts in SBS rats, and additionally increased when SBS rats were orally administered BSDL. Our study strongly supported the concept that the oral administration of BSDL could have potentially sustainable and long-term effects on the intestinal adaptive growth in the setting of SBS.

In conclusion, enteral BSDL supplementation up-regulates proliferation, reduces permeability, and induces Wnt/β-catenin signaling in intestinal epithelium, which exhibits a significant effect on the gut adaptive process in the rats following mSBR. These findings supported that BSDL-based therapies might be used to enhance adaptive intestinal growth in the patients with SBS. In the future, further research is still needed to identify the optimal dosage of enteral BSDL administration, and the mechanisms behind the activation of Wnt/β-catenin pathway.

## Supporting information

Supplementary Materials and Methods

## Abbreviations

APC: adenomatous polyposis coli
BSDL: bile salt dependent lipase
BT: bacterial translocation
CK-1: casein kinase-1
GAPDH: glyceraldehyde 3-phosphate dehydrogenase
GLP-2: glucagon-like peptide-2
GSK-3: glycogen synthase kinase-3
H&E: hematoxylin and eosin
ISC: intestinal stem cell
LeX: Lewis x
MLN: mesenteric lymph node
mSBR: massive small bowel resection
PN: parenteral nutrition
RIPA: radioimmunoprecipitation assay
SBS: short bowel syndrome
TJ: tight junction
